# Effect of different LED light colors on welfare, performance, some behavioral patterns, and blood parameters of Muscovy ducks

**DOI:** 10.1186/s12917-024-04200-x

**Published:** 2024-08-07

**Authors:** Eman Hefnawy, Eman Elgazzar, Ahmed Sabek, Saeed El-laithy, Souad Ahmed

**Affiliations:** Department of Hygiene and Veterinary Management, Faculty of Veterinary Medicine, Benha University, Moshtohour 13736, Benha City, 13518 Egypt

**Keywords:** Muscovy ducks, Light color, Behavior, Welfare, Fear reaction, Performance

## Abstract

**Background:**

The current study was conducted to assess the impact of different LED light colors on welfare indicators in Muscovy ducks. These welfare parameters encompassed growth performance, specific behaviors, tonic immobility (TI), feather score, haematological, and serum biochemical parameters. Eighty-four healthy unsexed Muscovy ducklings aged two weeks were randomly assigned to four groups (3replicates/group; each replicate contains 7 birds) based on different LED light colors. The first group was raised under white light, the second under red light, the third under blue light, and the fourth under yellow light. To assess the impact of various LED light colors on welfare, growth performance indicators (body weight, body weight gain, feed intake, and feed conversion ratio) were measured. Behavioral patterns including feeding, drinking, standing, walking, sitting, feather pecking, and other activities were recorded. Tonic immobility test (TI) and feather condition scoring were conducted at 3, 6, and 10 weeks of age. At the end of the study blood samples were collected for hematological and serum biochemical analyses.

**Results:**

The results revealed that using blue, yellow, and red colors had no adverse effect on the final body weight of the ducks (*P* > 0.05). Unlike to red light, blue light significantly reduced feather pecking, TI time and cortisol concentrations and improved the feather condition score (*P* ≤ 0.05).

**Conclusions:**

The current findings suggest that the application of blue light effectively improves welfare indices and has no detrimental impact on the growth performance of Muscovy ducks thereby positively contributing to their welfare.

## Background

The poultry industry plays an important role in national income of many countries. Ducks are mainly reared for meat and egg production. Thus making it one of the most significant industries helping to provide food security and economic development [1].

Muscovy ducks (Cairina moschata) hold a historical significance as they were among the first animals to be domesticated in South America. The domestication extended to Asia, Africa, and Europe, where they became popular in poultry farming due to their substantial size, rapid growth, and high slaughter weight [2–6]. The Muscovy duck is the main species utilized in duck farming and is a major resource for rural people in developing African nations like Egypt.

Light is considered one of the managerial factors affects poultry farming. The color, source, intensity, and duration of light affect the performance, behavior, welfare, and immunity of birds [7, 8]. Recently many countries have encouraged the use of light-emitting diode (LED) in poultry houses, LED bulbs are durable, have a wide range of wavelengths, produce low heat output, less energy consuming, and are available [9]. Birds are very sensitive to light; the brain receives information about environmental light through photoreception via routes from the pineal gland and retina of the eye [10]. Previous studies demonstrated the effects of different LED light colors on various poultry species as broilers [11–13], Mullard ducks [14], Pekin ducks [15], Brown Tsaiya ducks [16], and Cherry Valley ducks [17, 18]. Some birds prefer some light colors over others, performance and behaviors of broilers were improved under blue and green lights when compared to white and red lights [19]. According to previous reports blue, green, and yellow lights enhance the growth performance of broilers by improving the feed conversion ratio (FCR) [20], while the red light has negative impacts [21]. Combining blue and green lights improved the body weight, body weight gain, and FCR in broilers as blue-green light stimulates muscular buildup [11, 22].

A bird’s ability to deal with stressors and express behaviors can be influenced by light color [23]. The effect of light color on behavioral patterns is reported. The growth performance and immunity of broilers were affected by the blue light as it has a relaxing effect [24–26]. White and red light broiler groups were more active than blue and green light groups, while the red light group displayed greater aggression and floor pecking than the white, blue, and green light groups [19]. In Cherry valley ducks, blue and green lights decreased movement as ducks showed high setting frequency, while comfort behaviors were significantly performed in ducks raised under yellow light. Preening is one of the welfare indicator behaviors and is very essential for the birds. It was concluded that ducks under blue light spent longer time preening than ducks reared under white, yellow, and green lights [17].

Behavioral indicators like tonic immobility test (TI) and plumage score, physiology, and growth performance are mainly used to assess welfare, fear and stress in poultry [27, 28]. The long tonic immobility test duration indicates a great intensity of fear [29]. Fear reaction in some duck breeds as affected by LED light colors was previously reported by [14, 17] who reported the lowest TI durations in ducks raised under blue light. In comparison to hens raised under red and blue-green lights, hens in white and yellow-orange lights had a lower score for their backs feather. The yellow-orange group’s hens had the lowest score for rump plumage when compared to the other groups. For the tail feather score, there were no discernible changes between the light color treatments. Hens housed in the red and blue-green light groups improved the belly feather score compared to the white and yellow-orange light groups [30]. Blood profile and serum biochemical parameters are considered welfare measures that influenced by LED light colors in broilers [11]. Light color may cause stress for birds and there are many indicators that can be used to assess the stress.In pekin ducks, glucocorticoids (corticosterone and cortisol) contribute to stress reduction by keeping the internal environment of the body constant [31]. Housing broilers under blue or green light improved their immunity when compared to those housed under white or red light [32, 33]. Broilers under blue and blue- green lights had a significant increase in total protein levels, while there was no significant effect of light color on glucose concentrations [11].

Limited studies have evaluated the effects of different LED light colors on welfare indices including behavior, performance, and blood profile of ducks. Existing research primarily focuses on the fear response, with a notable absence of information on Muscovy ducks. Therefore, the current study was conducted to evaluate the influence of different LED light colors on the performance, behavior, welfare, and blood parameters of Muscovy ducks.

## Materials and methods

The study was conducted at the laboratory animals research centre, Faculty of Veterinary medicine, Benha University, Egypt. All research procedures were carried out in compliance with the recommendations of the guidelines for the care and use of animals. The protocol was approved by the Scientific Ethics Committee of Faculty of Veterinary medicine, Benha University, Egypt (BUFVTM 02–08–23).

### Birds and management

A total of 84 healthy unsexed Muscovy ducklings aged 2 weeks with an average body weight of 251.61 ± 3.23 g were purchased from a private local company in Egypt. The impacts of sex factors were disregarded because the purpose of this study was to assess the effects of light color on ducklings exclusively throughout the growing period. Birds were housed in four symmetrical pens; each measuring 3.5 meters in length, 3 meters in width, and 3 meters in height. The pens were previously cleaned and disinfected. A layer of chopped rice straw, about 10 cm, was evenly distributed over the floor of all pens before the arrival of ducklings. Each pen had an LED lamp of 9 watts fixed 2.5 m above the floor.

During the experimental phase, the average room temperature was 25.36 ± 0.30 °C, the relative humidity was 56.50 ± 2.97%, and the photoperiod was 23 hours light and 1 hour dark. All ducklings were vaccinated against avian influenza and fowl cholera at the age of 4 and 6 weeks, respectively. Feeders and drinkers were equally distributed in the pens, and clean, fresh water was available throughout the day. From 2 to 4 weeks old, a starter diet contained 22% crude protein was given [34], followed by a grower diet contained 19% crude protein as recommended by [35] from 5 to 10 weeks old.

### Experimental design

A total of 84 healthy unsexed Muscovy ducklings aged 2 weeks were randomly assigned to 4 groups according to different light-emitting diode (LED) light colors; each group contained 21 birds; each group was divided into 3 replicates with 7 birds per replicate. Each group was housed in a light-proof pen, which was divided into 3 parts using wooden barriers, one part per replicate. Each part’s dimensions were 2 m in length and 0.85 m in width. The birds in the first group were reared under white light; the birds in the second group were reared under red light; the birds in the third group were reared under blue light; and finally, the birds in the fourth group were reared under yellow light. The light cycle was 23 hours light and 1 hour dark with a light intensity of 5.66 ± 0.24 lx, and measured at the level of the bird´s back using a lux meter. The study was conducted during the growing period of Muscovy ducks, from 2 to 10 weeks old.

### Growth performance

At the end of the study, five birds from each replicate were selected randomly and weighted to obtain the final body weight (FBW). Feed intake was calculated weekly as the amount given to the birds per group minus the residual amount. The feed intake per bird in grams equals the amount of consumed feed divided by the number of birds. Body weight gain (BWG) equals final body weight minus initial body weight and feed conversion rate (FCR) equals feed intake/body weight gain.

### Behavioral observation

The behavioral observation started when the ducks were 3 weeks old, after giving them 1 week to adjust to light colors. Fifteen birds from each group (5 per replicate) were randomly selected and marked by leather leg bands for behavioral observation. The behavioral patterns of each group were recorded 3 days a week, twice per day, at 9.00–10.00 am and 2.00–3.00 pm. Each bird´s behavioral patterns were observed by focal observation for 3 min, with a total observation time of 15 min per replicate per group in the morning and in the afternoon. All observations were conducted by one observer who was present at all measurement points of the experiment. The distance between the observer and ducks was about 50 cm and the observer started observation 2 minutes after entrance to the pen. The ducks showed many behavioral patterns that can be categorized as consummatory behavior, which includes feeding and drinking. Sitting and standing are behaviors that are not engaged with any other activities. Locomotor behavior was represented by walking. Ducks showed some stereotypic behaviors such as litter scratching, object pecking, and head shaking. Comfort behaviors include preening, wing and leg stretching, and tail wagging. Aggression was recorded as feather pecking. Descriptions of these different behavioral patterns are displayed in Table 1.

**Table 1:** Descriptions of behavioral patterns of ducks

**Behavior**	**Description**
**Feeding**	The bird inserts its bill into the feeder to consume feed [36]
**Drinking**	The bird inserts its bill into the drinker to consume water [36]
**Standing**	The legs are in contact with the floor without any activity [34]
**Sitting**	Ducks lie on the ground with open or closed eyes [34]
**Walking**	The bird moves from one point to another without contributing to other activities [17]
**Litter scratching**	The bird scratches the floor by its leg [34]
**Object pecking**	Ducks peck the ground or other parts of the pen by their beak [17]
**Head shaking**	Complete lateral movement of the head [17]
**Preening**	The bird cleans its plumage using the beak [17]
**Wing and leg stretching**	The bird stretches the wing and the leg of the same side [17]
**Tail wagging**	The tail moves from side to side [17]
**Feather pecking**	The bird pecks, pulls or sometimes eats the feather of another individual [36]

### Tonic immobility test

To assess the fear response in birds as affected by different LED light colors. Nine ducks from each treatment were used in TI measurements at 3, 6, and 10 weeks old. The time of immobility (TI) was measured in seconds after each duck was placed in a separate testing chamber, inverted on its back and then restrained for 15 seconds, and then released and the observer stood 1.5–2.0 meters away to avoid eye contact. The recommended TI time was between 10 and 600 seconds. The trial was repeated if the duck was released for less than 10 seconds, as the fear reaction of birds tend to increase with longer TI.

### Feather condition score

The feather condition of 15 birds per group (5/replicate) was determined 3 times throughout the study period at 3, 6, and 10 weeks old using a scoring scale as follows: score 0: good, indicating full feathering; score 1: moderate, indicating slight feather pecking, slight damaged areas less than 1cm^2^; score 2: bad, indicating severe feather pecking, bleeding, sever damaged areas more than 2 cm^2^ [37].

### Blood sampling, hematological, serum biochemical, and hormonal analysis

At the end of the study, 9 ducks from each treatment (3 / replicate) were selected randomly and sacrificed for blood sampling. Ducks were held with their heads down and their wings and legs restrained to prevent vigorous movement. Slaughtering was done using a sharp knife that made a single cut across the neck, cutting the carotid arteries, jugular veins, oesophagus, trachea, and the connective tissues of the neck. Knife sharpness is very important during slaughtering birds without pre- slaughter anaesthesia or stunning as it promotes better bleeding and reduces discomfort and anxiety in birds by inducing rapid unconsciousness.

5 ml of blood was collected from each bird between 9.00 and 10.00 am into two clean, sterilized, and labeled different tubes. A total of 2 ml of blood was collected in a tube containing Ethylene-diamine-tetra-acetic acid (EDTA) anticoagulant to determine the total erythrocyte and leukocyte counts, hemoglobin, and differential leukocyte count. The heterophils-to-lymphocytes ratio (H:L) was calculated by dividing the number of heterophils by the number of lymphocytes, as described by [14]. The remaining 3 ml of blood was collected in a clean, sterilized, labeled tube without anticoagulant, and centrifuged at 3000 rpm for 15 min. The serum was separated and kept at -20 °C until further analysis. Serum biochemical parameters such as glucose, total protein, and albumin concentrations were measured according to [38]. Globulin concentration was measured by subtract albumin from total protein. Serum cortisol concentration was estimated as a stress indicator which previously evaluated in pekin ducks by Oluwagbenga et al. [31]. In the current study, cortisol was measured by ELISA using (Cortisol II, cobas®) kits according to the manufacturer’s recommendations.

### Statistical analysis

SPSS version 22 was used to analyze the data. Growth performance, behavioral patterns, welfare parameters, hematological, serum biochemical, and cortisol concentrations were analyzed using analysis of variance (ANOVA). The normality of the data distribution was evaluated by a Shapiro–Wilk test. Means and standard error means were used to present the data. *P* ≤ 0.05 was used to declare the data to be different.

## Results

### Growth performance

The results revealed that the effect of light colors on the final body weight of ducks was not significant. The highest final body weight was recorded for the white light group followed by yellow, red, and blue light groups respectively (Table 2).

**Table 2:** Effect of different LED light colors on growth performance of Muscovy ducks during growing period

**Parameter**	**LED light colors**				
	**White**	**Red**	**Blue**	**Yellow**	***P*-value**
**Initial body (g)**	252.00 ± 6.69	251.66 ± 6.69	250.33 ± 6.69	250.66 ± 6.69	0.99
**Final body weight (g)**	3277 ± 195.30	2989 ± 195.30	2844 ± 195.30	3056 ± 195.30	0.47
**Body weight gain (g)**	3025^a^ ± 57.73	2737^bc^ ± 57.73	2594^c^ ± 57.73	2805^b^ ± 57.73	0.005
**Feed intake (g)**	2910^a^ ± 57.73	2800^ab^ ± 57.73	2646^b^ ± 57.73	2914^a^ ± 57.73	0.03
**Feed conversion rate (FCR)**	0.96 ± 0.57	1.02 ± 0.57	1.02 ± 0.57	1.03 ± 0.57	0.5

Least square means (± SE) with different superscripts letters in the same row are significantly different at *p* ≤ 0.05

The number of the birds per groups was equal, each group had 21 ducks. The growth performance parameters were evaluated for 15 ducks per group

The effects of various LED light colors on body weight gain, feed intake, and feed conversion ratio (FCR) are displayed in Table 2. Birds raised under white and yellow light demonstrated higher feed intake (*P* = 0.03) and body weight gain (*P* = 0.005) than those reared under red and blue light color respectively. The current study revealed no effect of different light colors on body weight gain and feed conversion rate of ducks.

### Behavioral patterns

Behavioral frequencies as affected by different LED light colors are shown in Table 3. Feeding, drinking, sitting, walking, head shaking, and tail wagging were insignificantly influenced by LED light colors (*P* > 0.05). Standing was significantly affected by different LED light colors, as the highest frequency of standing was recorded in the red light group, followed by the white and yellow light groups, while the lowest standing frequency was observed in ducks reared under blue light (*P* = 0.008). There was a great impact of LED light colors on the comfort behaviors of ducks, as preening was higher in the red light group, white light group, and yellow light group than blue light group (*P* = 0.05). Ducks reared under white and red light groups showed more wing and leg stretch than ducks reared under blue, and yellow light respectively (*P* = 0.01). Stereotypic behaviors like litter scratching and object pecking were lower in blue and yellow lights than red and white lights. Feather pecking was lower in ducks reared under blue light than others (*P* = 0.05).

**Table 3:** Effect of different LED light colors on some behavioral patterns of Muscovy ducks during growing period

**Behavior**	**LED light colors**				
	**White**	**Red**	**Blue**	**Yellow**	***P*-value**
**Feeding**	2.76 ± 0.48	2.47 ± 0.48	1.95 ± 0.48	1.42 ± 0 .48	0.23
**Drinking**	4.76 ± 0 .92	4.47 ± 0.92	3.52 ± 0 .92	4.09 ± 0.92	0.80
**Sitting**	24.52 ± 1.41	22.38 ± 1.41	21.81 ± 1.41	21.61 ± 1.41	0.45
**Walking**	6.47 ± 0 .81	6.71 ± 0 .81	4.38 ± 0.81	4.71 ± 0.81	0.10
**Standing**	10.19^ab^ ± 1.34	13.57^a^ ± 1.34	6.85^b^ ± 1.34	9.71^ab^ ± 1.34	0.008
**Preening**	16.47^ab^ ± 1.80	17.09^a^ ± 1.80	11.57^b^ ± 1.80	14.09^ab^ ± 1.80	0.05
**Wing and leg stretch**	4.33^a^ ± 0.51	3.00^ab^ ± 0 .51	2.23^b^ ± 0.51	2.04^b^ ± 0.51	0.01
**Head shacking**	2.38 ± 0.48	2.95 ± 0.48	3.33 ± .048	2.95 ± 0 .48	0.58
**Tail wagging**	5.33 ± 0 .98	6.42 ± 0 .98	5.47 ± 0 .98	5.23 ± 0 .98	0.81
**Feather pecking**	3.19 ± 0.73^a^	3.38 ± 0 .73^a^	1.52 ± 0.73^b^	3.76 ± 0 .73^a^	0.05
**Litter scratching**	4.76^ab^ ± 0.75	5.66^a^ ± 0.75	2.52 ^b^ ± 0.75	3.14^b^ ± 0 .75	0.01
**Pecking object**	2.19 ^ab^ ± 0 .43	2.90 ^a^ ± 0.43	1.28^b^ ± 0 .43	1.52^b^ ± 0 .43	0.04

Least square means(± SE) with different superscripts letters in the same row are significantly different at *p* ≤ 0.05

The number of the birds per groups was equal, each group had 21 ducks. The behaviors were observed for 15 ducks per group

### Tonic immobility test (TI)

Table 4 shows the effect of several LED light colors on the fear reaction of ducks that are represented by TI. TI durations were significantly influenced by different LED light color treatments at different ages. At 3 weeks old the duration of TI was longer in ducks reared under blue, white, and red light than in ducks reared under yellow light (*P* = 0.05). At 6 weeks old, the red and blue light groups showed the greatest fear reaction with a TI duration of about 26.77 ± 2.90 and 18.55 ± 2.90 s respectively followed by the white, and yellow light groups, respectively (*P* = 0.01). At the age of 10 weeks, the fear reaction of birds was significantly affected by LED light color treatments, as birds reared under white and blue light colors displayed less fear reaction than birds reared under yellow and red light colors respectively (*P* < 0.001). The TI duration was 16.11 ± 2.22, 28.00 ± 2.22, 17.00 ± 2.22, and 33.00 ± 2.22 s for birds reared under white, red, blue, and yellow light respectively.

**Table 4:** Effect of different LED light colors on Fear reaction (tonic immobility test) (TI) of Muscovy ducks during growing period

TI test duration/ second	LED light colors				
	**White**	**Red**	**Blue**	**Yellow**	***P*-value**
**At 3 weeks old**	22.00^ab^ ± 2.53	19.22^ab^ ± 2.53	26.44^a^ ± 2.53	17.44^b^ ± 2.53	0.05
**At 6 weeks old**	16.00^b^ ± 2.90	26.77^a^ ± 2.90	18.55^ab^ ± 2.90	13.33^b^ ± 2.90	0.01
**At 10 weeks old**	16.11^b^ ± 2.22	28.00^a^ ± 2.22	17.00^b^ ± 2.22	33.00^a^ ± 2.22	< 0.001

Least square means(± SE) with different superscripts letters in the same row are significantly different at *p* ≤ 0.05

The number of the birds per groups was equal, each group had 21 ducks. The TI test was performed for 9 ducks per group

### Feather condition score

As shown in Table 5, at 3 weeks, ducks in all groups showed good feather condition (score 0). At 6 weeks ducks raised under yellow light displayed a moderate feather condition score (score 1) compared to birds in other groups (*P* < 0.001) which showed a good feather condition score (score 0). Finally at 10 weeks birds raised under white, red, and yellow light showed a bad feather condition score compared to birds raised under blue light that displayed moderate feather scoring (*P* = 0.005).

**Table 5:** Effect of different LED light colors on feather condition score of Muscovy ducks during growing period

Feather score	LED light colors				
	**White**	**Red**	**Blue**	**Yellow**	***P*-value**
** At 3 weeks old**	0	0	0	0	> 0.05
** At 6 weeks old**	0^b^	0^b^	0^b^	1^a^	< 0.001
** At 10 weeks old**	2^a^	2^a^	1^b^	2^a^	0.005

Scores with different superscripts letters in the same row are significantly different at *p* ≤ 0.05

The number of the birds per groups was equal, each group had 21 ducks. The feather condition score was performed for 15 ducks per group

### Hematological, serum biochemical, and hormonal parameters

Different LED light colors had no significant effect on hematological parameters, including red blood cells (RBCs), hemoglobin (HB), total leukocyte count (TLC), heterophils, lymphocytes, and heterophils: lymphocytes (H: L) ratio (*P* > 0.05). In contrast to the effect of LED light colors on hematological parameters, various LED light colors had a significant impact on serum biochemical parameters. Ducks reared under red light displayed the highest glucose concentrations, followed by ducks reared under white, yellow, and blue light respectively (*P* < 0.001). Birds raised under blue, yellow, and red light had the highest albumin concentrations compared to birds raised under white light (*P* = 0.001). Globulin concentrations were higher in white and red light groups than blue and yellow light groups (*P* = 0.01). Raising ducks under different LED light colors significantly affected their serum cortisol concentrations, as birds raised under blue light showed the lowest cortisol concentrations, while the highest concentrations were observed in ducks raised under red light, while other two groups (yellow and white) had intermediate concentrations (*P* < 0.001) (Table 6).

**Table 6:** Blood profile and serum biochemical parameters of Muscovy ducks during growing period as affected by different LED light colors

Items	LED light colors				
	**White**	**Red**	**Blue**	**Yellow**	***P*-value**
**RBCs × 10**^**6**^**/mL**	5.40 ± 0.12	5.50 ± 0.12	5.09 ± 0.12	5.36 ± 0.12	0.13
**HB (g/dL)**	15.76 ± 0.35	15.98 ± 0.35	14.83 ± 0.35	15.63 ± 0.35	0.13
**TLC × 10**^**6**^**/mL**	3.23 ± 0.16	3.26 ± 0.16	3.04 ± 0.16	3.11 ± 0.16	0.75
**Heterophils %**	15.00 ± 0.69	14.11 ± 0.69	16.00 ± 0.69	14.33 ± 0.69	0.24
**Lymphocytes %**	78.77 ± 0.85	80.33 ± 0.85	78.33 ± 0.85	80.55 ± 0.85	0.19
**H:L ratio**	0.19 ± 0.01	0.17 ± 0.01	0.20 ± 0.01	0.17 ± 0.01	0.23
**Glucose (mg/dL)**	175.88^b^ ± 6.39	207.22^a^ ± 6.39	117.66^c^ ± 6.39	171.66^b^ ± 6.39	< 0.001
**Albumin (g/dL)**	1.93^b^ ± 0 .11	2.15^ab^ ± 0.11	2.63^a^ ± 0.11	2.48^ab^ ± 0 .11	0.001
**Globulin (g/dL)**	2.43^a^ ± 0.14	2.34^a^ ± 0.14	1.86^b^ ± 0 .14	1.87^b^ ± 0 .14	0.01
**Total protein (g/dL)**	4.36 ± 0 .08	4.51 ± 0 .08	4.50 ± 0 .08	4.36 ± 0 .08	0.44
**Cortisol (ug\dL)**	1.72^b^ ± 0.14	2.87^a^ ± 0.14	0.31^c^ ± 0 .14	1.91^b^ ± 0 .14	< 0.001

Least square means (± SE) with different superscripts letters in the same row are significantly different at *p* ≤ 0.05

The number of the birds per groups was equal, each group had 21 ducks. The Blood profile and serum biochemical parameters were performed for 9 ducks per group

## Discussion

### Growth performance

The results revealed that ducks under blue light showed a lower final body weight than other groups. However, this reduction in body weight was not significant. Other managemental factors than light color may affect the body weight of birds, as in the current study the feeding management is the same between the different treatment groups. The same effect of light color was reported in broilers as chickens reared under blue light showed the lowest body weight at the end of the experiment when compared to those reared under red, green and yellow light however the difference between the body weight of broilers in blue, red, and green groups was not significant [39]. The outcome supports the claim that broilers’ final body weight was not significantly affected by the different light colors (white, red, green, and blue) [19]. The result also agrees with [40] who found no significant effect of different light color treatments (yellow, red, green, and blue) on the body weights of Fayoumi chickens. Light had no impact on broilers body weight [41]. There was no impact of light colors on body weight of Pekin ducks throughout the study period (1–42) days [15]. In contrast, broilers reared under blue light showed a significant heavier body weight (*P* = 0.001) than those reared under white light [11]. At 42 days, the final body weight of Cherry Valley ducks reared under blue and green light was higher than the final body weight of ducks reared under white and yellow [18]. Our findings in contrast with [26] who reported that broilers raised under blue light were heavier than those under white light. While the number of meals and the amount of feed were the same in all groups, the different LED light color treatments significantly affected body weight gain and feed intake. Compared to other groups, the white and yellow light groups’ body weight increase was noticeably larger, which could be explained by the fact that these groups of ducks consumed feed more frequently. Parallel to the body weight gain, ducks reared under white and yellow light displayed the highest feed intake rates when compared to those reared under red and blue light respectively. Our results agree with [24] who reported that yellow light had a positive impact on growth performance of broilers by improving growth. In line with the current findings, Pekin ducks reared under white and red lights gained more body weight than ducks reared under blue light [42]. In the current study, FCR was not significantly influenced by different light color treatments. The result agrees with [18] who reported that different light colors had no effect on FCR of ducks. Unlike to the current result, performance parameters including body weight gain, feed intake and FCR were significantly enhanced in broilers raised under blue light than those reared under white light [11]. The light color had no effect on feed intake and body weight gain of chickens [18, 40, 41] did not report any effect of different light colors in feed intake of ducks along the experimental period.

### Behavioral patterns

The study aims to detect the effect of different LED light colors on some behavioral patterns in ducks. The different light color treatments had no significant effect on consummatory behavior (feeding and drinking) which may be attributed to the fact that all birds have the same light intensity, and by adapting to the room light color, birds are able to find feeders and drinkers easily to obtain food and water. Our findings concur with the previous study on Pekin ducks that found no variation in feeding frequency as influenced by different light colors [42]. The feeding time of chickens was not affected by rearing under different light colors [19, 43] reported no significant impact of light colors on the feeding and drinking of broiler chickens. Contrary to the current result, feeding behavior increased in broilers reared under red and green light [44] or blue light [40].

Behaviors that are not engaged with activities are sitting and standing. In the current study, it is consistent that sitting bouts were not significantly affected by different light color treatments while standing frequency increased significantly in birds raised under red light compared to those reared under white, yellow, and blue light, respectively. This may be due to the association between red color and negative emotions such as fear and danger that keep the birds standing most of the time. Unlike our result, chickens reared under red light had the shortest standing times compared to those reared under white, green and blue light however this difference was not significant [19]. Similarly, there was no impact of different light colors (white, blue, and green) on the standing behavior of broilers, as mentioned by [12]. In contrast to the current findings, blue light reared ducks spent longer time setting than those reared under yellow light while, there was no difference in sitting between ducks reared under blue light and those reared under white and green light [17]. The variation observed between the current result and previous studies on the effect of different LED light colors on inactive behaviors may be attributed to species differences. Although different light treatments had no significant impact on walking behavior, ducks reared under red and white light showed more walking frequency than ducks reared under yellow and blue light. Thus, it may occur due to the longer wavelengths of red and white colors that pass effectively through the brain and retina of birds, making them more active. The result agrees with [40, 43] who reported that walking duration and frequency was higher in broilers raised under white and red light than those raised under blue, yellow, and green light. The light color of the duck considerably affected its walking behavior. Walking activity was dramatically reduced by the blue light [17]. In the current study, head shaking remained unchanged between the different light color treatments, this was consistent in the previous study conducted by [17]. Litter scratching and object pecking including ground pecking were significantly affected by LED light colors; the highest frequencies of litter scratching and object pecking were recorded in birds reared under red light, while the lowest frequencies were recorded in birds raised under blue light, while the white and yellow light groups were intermediate. Red light may stimulate the extra retinal photoreceptors which located in the hypothalamus that control most of duck activities including litter scratching and object pecking. Chickens raised in blue light were less likely to engage in ground pecking, with white light being intermediate [19]. The result agrees with [43] who reported higher frequency of ground pecking in broilers reared under white light than those reared under blue light. Ground pecking was lower in ducks raised under blue light than ducks reared under white, green, and yellow light specially at morning while during afternoon ground pecking was significantly lower in blue light group than yellow light group [17]. As the same to our findings [40], found that chickens reared under red light showed more litter scratching than those raised under blue and yellow light. Laying hens under green light showed higher object pecking behaviors than those raised under red and white color, respectively [45].

Comfort behaviors showed a variation in their response to different light colors. Preening and wing and leg stretching were significantly changed as affected by light color while tail wagging remained unchanged. Preening and wing and leg stretching increased in ducks reared under white and red light comparing to birds reared in blue and yellow light groups. Preening and stretching may be increase in red and white light groups due to the stimulating effects of these colors that improve birds’ activities while blue color has a relaxing impact. The percent of preening behavior of Brown Tsaiya ducks was 30%, 26.36%, and 24.5% for white, red, and blue light colors respectively [16]. Contrary to the current result, preening and stretching frequencies were higher in broilers [12] and Cherry Valley ducks [17] raised under blue light than those reared under white light [19] stated that wing stretching was less common in chickens reared under blue light and more common in birds reared under red and white lights. The light color had no significant effect on comfort behaviors of broilers as described by [43]. In the current study, aggression was represented by feather pecking, which showed the lowest bouts in birds under the blue light when compared to other birds under the yellow, red, and white groups respectively. This may be attributed to the short wavelength of the blue light, which calms the birds and decreases the incidence of feather pecking. The aggression interaction was higher in chickens reared under red and white light than those reared under blue light however this increase was not significant [19].

### Tonic immobility test (TI)

There are some indices that can be used to evaluate fear response in birds like immobilization, number of vocalizations, frequency of elimination, and body shaking [14]. In the current study fear response in ducks was evaluated by using tonic immobility test (TI). The results revealed that TI duration in the 3rd week of age was longer in ducks reared under blue light than those reared in white, red, and yellow light treatments respectively however, the difference between the blue, white, and red light groups was not significant. These findings were changed at the 6th and 10th week of age, at 6 weeks old the shortest TI duration was recorded in yellow light group while the longest duration was reported in the red light. At the 10th week the fear response was higher in yellow and red light treatments than blue and white light respectively. The fear response was higher at 3 weeks age in ducks under blue color than other ducks which may be due to ducks still not adapted to the light treatments after that with adaptation the blue color starts to show its calming effect on ducks which represented with TI short durations at 6 and 10 weeks old. Increase the fear response in ducks raised under yellow light at the end of the study may be attributed to creation of stress and danger feeling of yellow light but this result needs more investigations. The results agree with [12] who reported that birds under blue light had a short TI latency. Unlike [17] the current result reported the shortest TI duration in ducks under yellow light at 6 weeks age [43] reported no effect of light color on TI of broilers. There was a significant effect of light color on TI duration. The broilers under blue light treatment showed the significant lowest TI duration compared to those raised under white light [11].

### Feather condition score

There is a shortage in studies regarding the effect of light colors on feather condition in ducks, the current finding revealed that the feather condition of ducks reared under blue light tended to be superior to those reared under other light color treatments. These results may be attributed to the calming effect of blue light that decreases the incidence of feather pecking while keeping good feather condition. The result agrees with [30] who reported that the overall plumage condition score in hens reared under blue green light was better than those reared in white, and yellow orange color treatments.

### Haematological, serum biochemical parameters, and cortisol concentrations

Several previous studies demonstrated the effect of light on haematological, serum biochemical, and cortisol concentrations in different poultry species [11, 14, 31, 39, 42, 46]. In the present study, RBCs count, total leukocytic count, heterophils count, lymphocytes count, haemoglobin concentration, and H:L ratio were unaffected by different LED light color treatments. Ducks under red light showed the highest glucose and cortisol concentrations, while the lowest concentrations were reported in the blue light group, with birds in white and yellow colors situated in between, which may reveal the stressful effect of the red light color on birds. Albumin and globulin were significantly affected by light color, as albumin concentrations were higher in birds under blue and yellow light than those under red and white light. Unlike globulin concentrations, which were higher in ducks under red and white light than in blue and yellow light, Birds under blue light showed the lowest cortisol concentrations compared to birds under red light, which displayed the highest cortisol concentrations. This study’s findings on cortisol levels and the H: L ratio revealed that blue light did not cause stress in ducks, as previously reported in broilers by [43]. Raising broilers under the blue light alleviated stress [47]. Unlike the current findings, different light colors had no significant effect on serum glucose, albumin, and globulin concentrations in Cherry Valley ducks [18] while in Pekin ducks, it was stated that birds reared under blue light had higher plasma corticosterone concentrations than those reared under red and white light [42]. In Mullard ducks, the effect of different LED light colors was significant on red blood cells, leucocyte count, and H:L ratio, while hemoglobin concentration was unaffected by light color, and ducks reared under blue light showed lower plasma corticosterone than those reared under red and white light [14]. In broilers, the blue light increased the red blood cell count and hemoglobin concentrations compared to white light, while TLC and H:L ratios were superior in broilers under white light to those under blue light [11].

## Conclusion

In conclusion blue and yellow light had no negative effects on Muscovy ducks’ final body weight. The short durations of TI, which indicate a mild anxiety and fear reaction and low cortisol concentrations, indicate that blue light did not produce any physiological stress in the ducks. When compared to other light color treatments, Muscovy ducks raised under blue light demonstrated the lowest frequency of feather peaking and the best feather condition score. In the Muscovy ducks’ sector, blue light is strongly recommended. However, the absence of sex differentiation is a limitation of the current study and further research is needed to determine the effects of sex on ducks reared under different light colors. 

## Data Availability

The data presented in this study are available within the article.
